# A portrait of germline pathogenic variants in high and moderate penetrance breast cancer genes in Brazil

**DOI:** 10.3389/fonc.2024.1495605

**Published:** 2024-12-17

**Authors:** Leandro Jonata de Carvalho Oliveira, Amanda Muniz Rodrigues, Carolina de Bustamante Fernandes, Fernanda Orpinelli Ramos do Rego, Fernanda Christtanini Koyama, Andreza Karine de Barros Almeida Souto, Thaiana Aragão Santana, João Paulo Gonzaga de Faria, Marcela Lima Bulcão, Ivana Lucia de Oliveira Nascimento, Ana Carolina Branco Neves Silva, Isabela Pessoa Elias Gonçalves, Rayana Elias Maia, Renata Gondim Meira Velame de Azevedo, Layla Testa Galindo, Daniela Vianna Pachito, Adriana Cury, Mariano Gustavo Zalis, Bruno Lemos Ferrari, Bernardo Garicochea, Rodrigo Dienstmann

**Affiliations:** ^1^ Oncoclinicas&CO – Medica Scientia Innovation Research (MedSir), São Paulo, Brazil; ^2^ Oncoclinicas (OC) Medicina de Precisão (OCPM), São Paulo, Brazil; ^3^ Pfizer Brasil, São Paulo, Brazil

**Keywords:** hereditary breast cancer, cancer genetics, germline genetic testing, multigene panel testing, Brazil

## Abstract

**Introduction:**

The prevalence of germline pathogenic/likely pathogenic variants (P/LP) in high and moderate penetrance (HMP) genes is approximately 7%–10% among breast cancer (BC) patients. The prevalence and spectrum of BC P/LP variants are affected by several factors. There are limited genetic data from Brazilian patients with BC.

**Methods:**

This is a retrospective cross-sectional study that aims to evaluate the germline profile of P/LP variants in 13 HMP BC genes (*BRCA1, BRCA2, PALB2, TP53, CDH1, NF1, PTEN, STK11, CHEK2, ATM, BARD1, RAD51C*, and *RAD51D*) in patients diagnosed with BC in Brazil. All patients were tested using multigene NGS panels covering from 35 to 105 genes. Primary endpoint was the prevalence of P/LP variants in BRCA1/2 and in other HMP genes. Secondary endpoints were stratified analyses according to age and BC subtype.

**Results:**

This cohort involved 2,208 patients with BC from 2019 to 2023. Most patients (79.7%) were from Southeastern Brazil. The median age at genetic testing was 47 years, and most patients (59.4%) were ≤50 years. The BC subtype was available in 641 cases: 264 patients (41.2%) were HR+/HER2−, 116 (18.1%) were HER2+, and 261 (40.7%) had triple-negative breast cancer (TNBC). Overall, 215 (9.7%) had a P/LP in HMP genes, including 5.8% in *BRCA1/2*. The most frequent variants were found in *BRCA2*, *BRCA1*, and *TP53*. The founder variant R337H accounted for 79% of all *TP53* pathogenic variants, representing 1% of the overall population. Deleterious variants in *BRCA1/2* were more common in patients ≤50 years (7.7%) and TNBC (10.7%). In other HMP BC genes, the prevalence of P/LP variants did not significantly vary according to age and BC molecular subtype. The overall VUS rate in HMP genes was 19.6%.

**Conclusion:**

In Brazil, the epidemiology of deleterious variants in HMP is comparable to published US and EU cohorts. The Brazilian *TP53* R337H is a prevalent variant in BC patients. Deleterious *BRCA1/2* variants vary according to age and BC subtype. Our study gives a broader understanding of BC risk genes and has opened doors to optimized testing and surveillance strategies in Brazil.

## Introduction

Breast cancer (BC) is the most common type of cancer among women worldwide and also the leading cause of cancer-related mortality—accounting for an estimated 2,268,333 new cases and 660,620 deaths in 2022 (1). In Brazil, 73,000 new cases of female BC were estimated in 2023, representing 30% of all neoplasms, and 17,000 deaths occurred in 2020 (2). Hereditary breast cancer (HBC) accounts for approximately 10% of all cases, of which approximately 50% are due to germline variants in *BRCA1/2* (3, 4). Next-generation sequencing (NGS) panel tests have identified mutations in other cancer-associated genes in BRCA1/2-negative patients with suspected HBC, sometimes more than doubling the mutation detection rate (5–9). This technology has dramatically expanded the scope of HBC—other high and moderate penetrance (HMP) genes are becoming of increased relevance—and speed of genetic testing with reduced cost (10).

Worldwide, the estimated prevalence of germline pathogenic/likely pathogenic variants (P/LP) in HMP genes varies between 5% and 13% among women with BC in Caucasian-based studies (3–6, 8, 9). The prevalence and spectrum of BC P/LP variants are affected by age at diagnosis, race/ethnicity, ancestry, geographical region, and BC molecular subtype (9, 11, 12). A cross-sectional study that evaluated the prevalence of P/LP and variants of unknown significance (VUS) among individuals undergoing NGS panel testing for Hereditary Breast and Ovarian Cancer (HBOC) from Mexico, Central America, the Caribbean, South America, and self-reported Hispanic individuals from US showed an LP/P rate ranging from 9.1% to 18.3%. The South America rate of P/LP and VUS were 13.8% and 40.6%, respectively (13).

In Brazil, there are some published studies evaluating the prevalence of other HMP genes beyond BRCA1/2 with multigene panel testing (14–21). The prevalence of germline findings varies widely, depending on the selected demographic, clinical, and pathological factors as well as the number of genes included in the panel, some of which incorporate low-penetrance genes and non-HBC genes (14, 17, 20). One large study showed the prevalence of 17.5% in HMP genes in Brazilian BC patients tested in a single laboratory (21). However, the prevalence of HBC genes according to BC subtypes remains largely unexplored in Brazil. Many international cohorts revealed significant molecular heterogeneity for predisposition genes within BC subtypes (5, 22).

The aim of this study is to evaluate the prevalence of P/LP variants and VUS in *BRCA1/2* as well as in other HMP BC genes, in the overall population diagnosed with BC referred to a central laboratory. In addition, we aimed to perform stratified analyses according to age and BC subtype.

## Methods

### Study design and population

This is a retrospective cross-sectional study that evaluates the germline profile of patients diagnosed with invasive BC (ICD-10 code C40) from 12 different sites in South, Southeast, Midwest, Northeast, and North regions of Brazil and were tested in a single reference laboratory [Oncoclinicas (OC) Medicina de Precisão (OCPM), São Paulo, Brazil] from 2019 and 2023. OCPM is a reference laboratory to the Oncoclinicas & CO group, which is the largest private healthcare provider of oncology care in Latin America. This study was approved with waiver of re-consent by the local Research Ethics Committee (CAAE: 70500223.8.0000.0227) in Rio de Janeiro.

Patients underwent testing with either a targeted HBOC panel of 35 genes or a broader germline panel covering 105 genes, at the discretion of the physician. Both assays cover HMP BC genes: *BRCA1, BRCA2, PALB2, TP53, CDH1, NF1, PTEN, STK11, CHEK2, ATM, BARD1, RAD51C*, and *RAD51D*. Irrespective of the panel, NGS followed the same protocols. Genomic DNA was obtained from a buccal swab or peripheral blood sample. NGS libraries and panel enrichment was prepared using SureSelect custom panel XTHS2 (Agilent). DNA sequencing was performed by Illumina platforms (NextSeq 550). The bioinformatic pipeline consisted of FastQ files (generated by Illumina’s pipeline) aligned to the reference genome GRCh37/UCSC hg19, low-quality and duplicate readings removal, and variants (SNPs/indels) calling with GATK HaplotypeCaller on the coding sequences and flanking regions (± 20 bp) of the target region. Copy number variations (CNVs) were identified at the exon level using both ExomeDepth and CNVkit. If a CNV was identified, a multiplex ligation-dependent probe amplification (MLPA) assay was performed as orthogonal confirmation. The variants were manually classified by internal molecular biologists and geneticists according to the guidelines of the American College of Medical Genetics and Genomics (ACMG)/Human Genome Variation Society (HGVS) considering the current literature (23).

### Clinical database

Patient data, such as gender, age at testing, geographic region, and diagnosis of BC, were obtained from mandatory test requisition forms filled by ordering physicians and structured in the laboratory information management system (LIMS). The BC molecular subtype was also extracted from local LIMS for the subset of patients with information on hormone receptor (HR, both estrogen and progesterone) and HER2 status by immunohistochemistry (IHC) and fluorescence *in situ* hybridization (FISH). BC samples were classified into three subtypes, HR+/HER2−, HER2+, or triple-negative breast cancer (TNBC), as per standard practice. All data were anonymized before analysis.

### Statistical analysis

Descriptive statistics were used to summarize the data. Categorical data were presented as frequency and percentages, and continuous data were expressed as medians and ranges. For comparisons of categorical and continuous variables, we used the chi-square test and Mann–Whitney U test, respectively. Statistical significance was assumed at *p* < 0.05. As the study was descriptive, estimation of sample size or statistical power was not applicable. All data were processed in Microsoft Office Excel along with R Programming Environment, version 4.0.5.

## Results

### Clinical and molecular characteristics

This cohort involved 2,208 patients with BC from 2019 to 2023. Most patients were women (99%) from Southeastern Brazil (79.7%), followed by patients from the Midwestern (5.6%), Southern (5.0%), and Northeastern/Northern (4.8%) parts. The median age at genetic testing was 47 years; most patients (59.6%) were ≤50 years.

BC molecular subtype was available in 641 cases: 264 patients (41.2%) had HR+/HER2− BC, 116 (18.1%) were HER2+, and 261 (40.7%) had TNBC (**Table 1**).

**Table 1:** Baseline characteristics.

	Overall cohort *N* = 2,208 (100%)	BRCA1/2 P/LP *N* = 129 (5.8%)	Other HMP genes P/LP *N* = 86 (3.9%)
Age at GT			
≤35 years	261	22 (8.5%)	17 (6.5%)
≤40 years	594	43 (7.2%)	35 (5.9%)
≤50 years	1,317	101 (7.7%)	57 (4.3%)
≤65 years	1,936	123 (6.3%)	77 (3.9%)
>50 years	891	28 (3.1%)	29 (3.2%)
>65 years	272	5 (1.8%)	10 (3.6%)
Median age	47 (19–95)	43	45
Gender			
Male	21	2 (9.5%)	1 (4.8)
Female	2,187	127 (5.8%)	86 (3.9%)
BC subtype			
HR+/HER2−	264	11 (4.2%)	7 (2.6%)
HER2+	116	4 (3.4%)	5 (4.3%)
TNBC	261	28 (10.7%)	8 (3.1%)
Not available	1,567	88 (5.6%)	65 (4.1%)
Region of Brazil			
North/Northeast	105	12 (11.4%)	3 (2.8%)
Midwest	123	7 (5.7%)	3 (2.4%)
Southeast	1,761	96 (5.4%)	73 (4.1%)
South	111	9 (8.1%)	6 (5.4%)
Not available	108	5 (4.6%)	2 (1.8%)

GT, germline testing; BC, breast cancer; HR+, hormone receptor positive; TNBC, triple-negative breast cancer; P/LP, pathogenic and likely pathogenic; HMP, high and moderate penetrance.

### Prevalence and spectrum of pathogenic and likely pathogenic variants

Overall, 215 (9.7%) had a P/LP in HMP genes, including 129 (5.8%) patients who had a *BRCA1/2* P/LP variant and 86 (3.9%) who had a P/LP variant in other HMP BC genes (**Table 1**). The prevalence of *BRCA1/2* P/LP variants was significantly higher in patients ≤50 years than in those >50 years (7.7% vs 3.1%; *p* = 0.009) as well as in those with TNBC (10.7%) when compared to HR+/HER2− (4.2%) and HER2+ (3.4%) (*p* < 0.001). **Table 2** summarizes these results.

**Table 2:** Prevalence of germline mutations in selected subgroups of interest.

	Age		Molecular subtype		
	≤50 years	>50 years	HR+/HER2−	TNBC	HER2+
BRCA1/2 P/LP	101 (7.7%)	28 (3.1%)	11 (4.2%)	28 (10.7%)	4 (3.4%)
Other HMP genes P/LP	57 (4.3%)	29 (3.2%)	7 (2.6%)	8 (3.1%)	5 (4.3%)
Wild type for P/LP	1,159 (88%)	834 (93.7%)	246 (93.2%)	225 (86.2%)	107 (92.3%)
*p*-value	*p* = 0.009		*p* < 0.001		

HR+, hormone receptor positive; TNBC, triple-negative breast cancer; P/LP, pathogenic and likely pathogenic; HMP, high and moderate penetrance.

**Figure 1:**
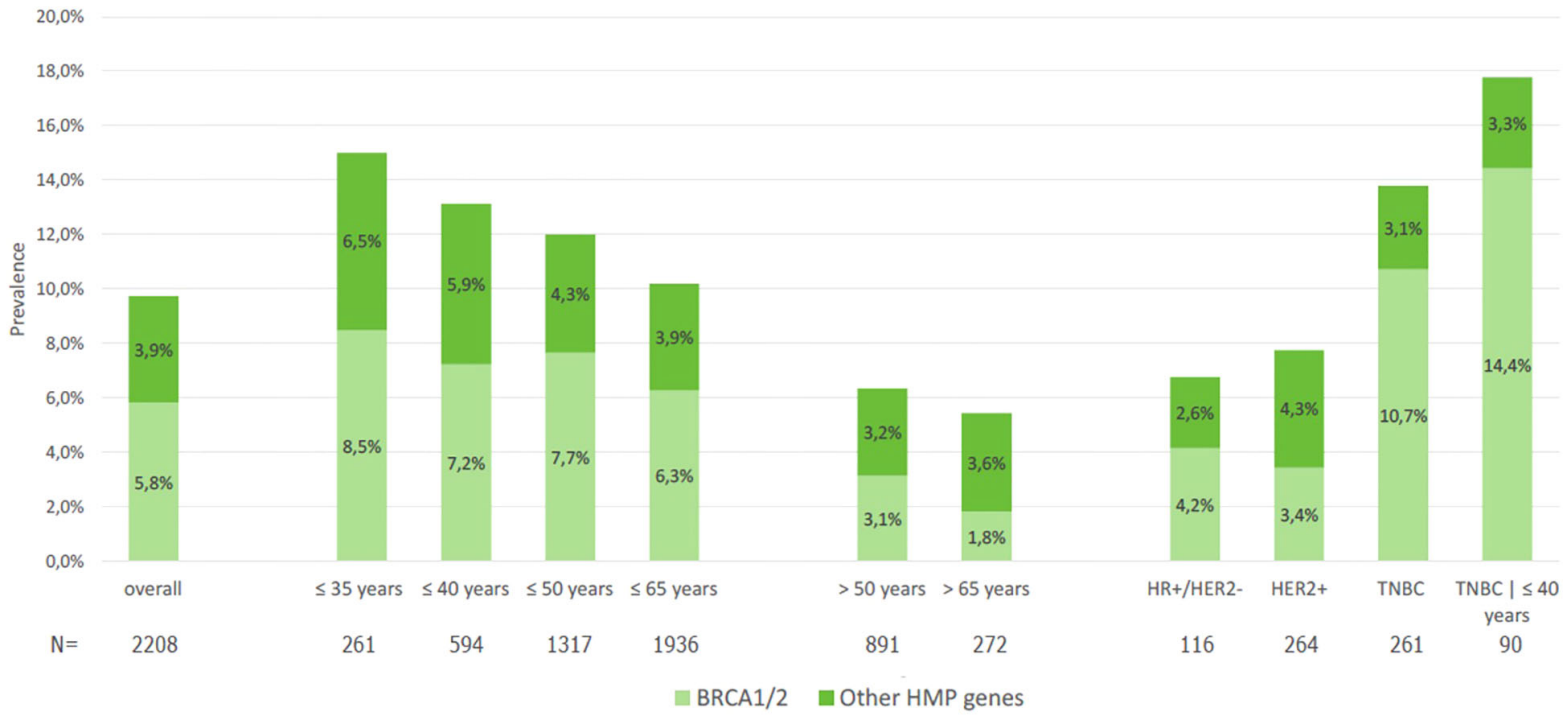
Prevalence of pathogenic or likely pathogenic variants in BRCA1/2 and other HMP genes in the overall population and according to age and breast cancer subtype.

**Figure 2:**
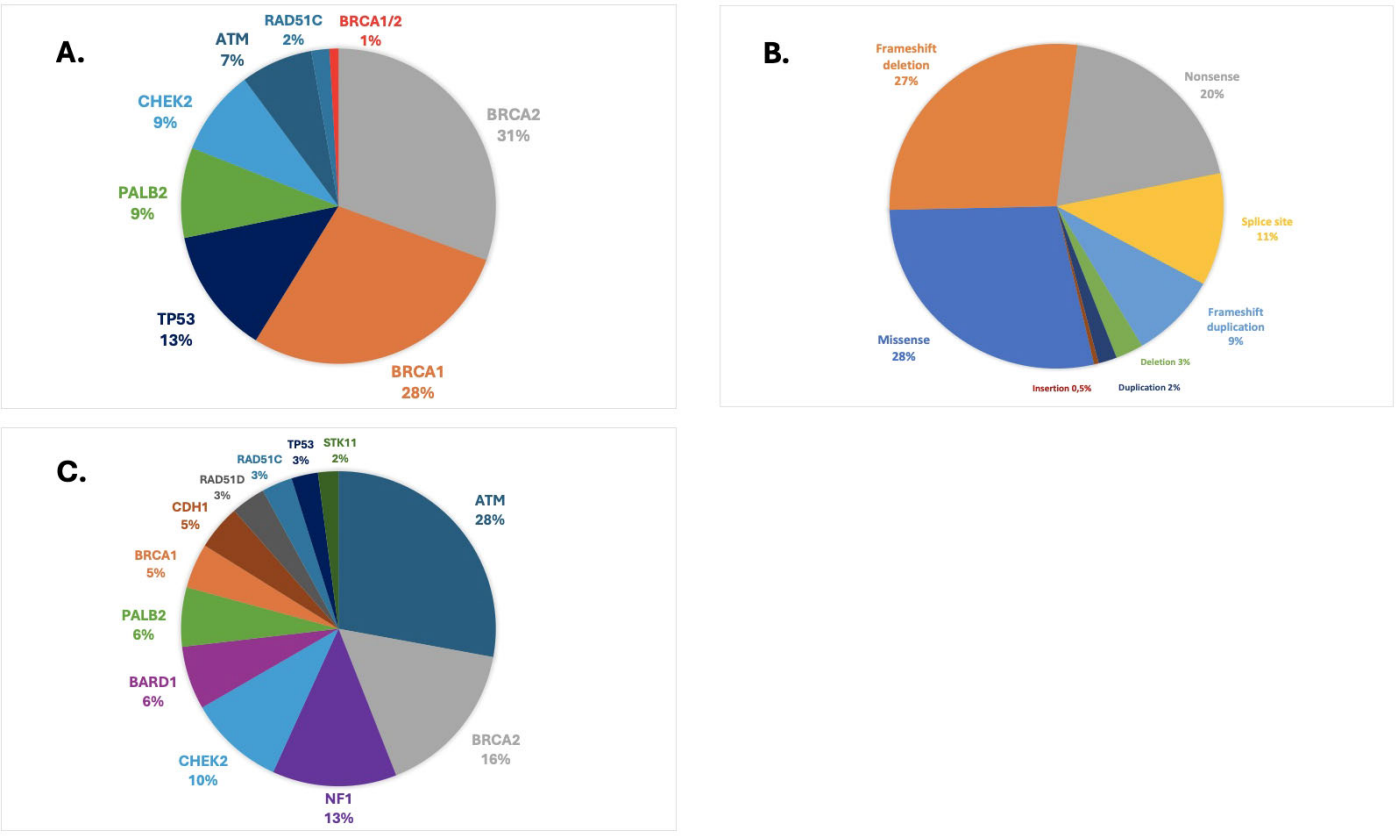
**(A)** Prevalence and spectrum of pathogenic or likely pathogenic variants in HMP genes. **(B)** Class of pathogenic or likely pathogenic variants. **(C)** Prevalence and spectrum of VUS in HMP genes.

Of note, patients with TNBC who were 40 years or younger had 14.4% of deleterious variants in *BRCA1/2* (**Figure 1**). In the population ≤65 years, per the new ASCO guideline cutoff for genetic testing recommendation in BC, the prevalence of *BRCA1/2* P/LP variants was 5.6%, compared to 1.8% in those >65 years. Two patients (1%) had two P/LP variants involving a combination of *BRCA1* and *BRCA2* genes and were diagnosed with MINAS (Multi-locus Inherited Neoplasia Allele Syndrome) (**Figure 2**).

On the other hand, the prevalence of deleterious variants in other HMP genes (excluding *BRCA1/2*) was 3.9% and did not significantly vary according to age categories (**Table 1**), although we observed numerically higher rates of P/LP variants in patients ≤35 years (6.5%) than in those >50 years (3.2%) or >65 years (3.6%) (**Figure 1**). In terms of BC molecular subtypes, the prevalence of P/LP variants in other HMP genes (excluding *BRCA1/2*) was 2.6% in HR+/HER2−, 4.3% in HER2+, and 3.1% in TNBC. Finally, among 21 male patients with BC, 2 (9.5%) had P/LP variant in *BRCA2* and 1 in *CHEK2* (4.8%), accounting for 15.3% of actionable findings.

The most frequent deleterious variants were found in *BRCA2* (31%), *BRCA1* (29%), *TP53* (13%), *PALB2* (9%), and *CHEK2* (9%) (**Figure 2A**). The founder pathogenic variant in *TP53* R337H accounted for 79% (22/28) of all *TP53* variants, representing 1% of the overall BC population included in this study. We did not find P/LP variants in *CDH1, NF1, PTEN, STK11, BARD1*, or *RAD51D*. In the HR+/HER2− population, the most frequently mutated genes harboring P/LP variants were *BRCA2* (33%), *BRCA1* (27%), and *PALB2* (22%). In HER2+ cases, these genes were *BRCA2* (33%) and *TP53* (22%). In patients with TNBC tumors, deleterious variants were most commonly found in *BRCA1* (58%), *BRCA2* (19%), or *PALB2* (17%). The largest fraction of deleterious variants were missense variants (28%), followed by frameshift deletion (27%), nonsense (20%), splice site (11%), and frameshift duplications (9%). CNVs such as large deletions and duplications accounted for 5% of all variants (**Figure 2B**).

### Prevalence and spectrum of variants of unknown significance

Overall, we found 495 VUS in HMP genes among 433 patients (19.6%). At least one VUS in *BRCA1/2* was detected in 4.4% of the cases, while 15.9% of the patients carried at least one VUS in other HMP genes. The most frequent VUS were found in *ATM* (28%), *BRCA2* (16%), *NF1* (13%), and *CHEK2* (10%) genes (**Figure 2C**). In the population who carried P/LP *BRCA1/2* variants, 17% had at least one VUS in HMP genes.

## Discussion

To the best of our knowledge, this is the largest study to investigate a cohort of Brazilian patients with BC from the private healthcare system who received multi-gene NGS panels in the single reference laboratory and may not have been strictly selected for germline genetic testing (GGT) based on high-risk criteria for hereditary cancer [National Comprehensive Cancer Network (NCCN)]. We found that the prevalence of P/LP variants in 13 HMP genes was close to 10%, including 6% of deleterious variants in *BRCA1/2* genes, which were significantly more prevalent in younger patients and in those with TNBC.

In Brazil, most published studies evaluating the prevalence of germline findings in BC patients were enriched for high-risk criteria for hereditary cancer—including low-penetrance genes and non-BC genes—and the prevalence varies between 15% and 20% (16–18, 20, 21). In the largest previous study, Guindalini et al. evaluated 1,663 Brazilian BC patients, who underwent germline multi-gene panels covering from 20 to 38 genes, which showed a 17.5% rate of deleterious variants in HMP genes, including 10.1% in *BRCA1/2*, 13.4% in high-penetrance (HP), and 4.1% in moderate-penetrance (MP) genes. Most mutated genes were *BRCA1* (27.4%), *BRCA2* (20.3%), and *TP53* (10.5%) in the HP group and *ATM* (8.8%) and *CKEK2* (6.2%) in the MP group. Of note, the R337H variant accounted for 70% of all *TP53* pathogenic variants, representing 1% of the overall population (21). The worldwide prevalence of germline *TP53* deleterious variants is estimated to be approximately one in every 3,500 to 10,000 individuals (24). However, in Brazil, a TP53-R337H founder PV in the South and Southern regions has a prevalence of approximately 0.3% of the healthy individuals (25). Among Brazilian women with BC, the prevalence of TP53 R337H varied from 0.9% to 12%, depending on the geographical region and age at diagnosis of BC (16, 21, 26–28).

Our results demonstrated lower prevalence of actionable germline findings, which could be explained by patient selection for NGS panel testing. Guindaline et al. selected for high-risk population as it included many patients referred to GGT in a laboratory (Mendelics Análise Genômica S.A., São Paulo, SP, Brazil) where testing costs could be reimbursed based on the restrictive coverage criteria (i.e., very high-risk criteria for GGT) defined by the Brazilian National Supplementary Health Agency (ANS—Agencia Nacional de Saude). In our study, all patients were tested in a single reference laboratory (OCPM, São Paulo, Brazil) using germline multigene panels where currently testing costs cannot be covered by health insurance and the GT recommendation was based on physician recommendation and out-of-pocket payment. Therefore, our study may have shown the prevalence of germline variants in a scenario closer to universal GGT as proposed by the American Society of Breast Surgeons (ASBrS) (29). According to the American Society of Clinical Oncology (ASCO) and Society of Surgical Oncology (SSO) guideline, *BRCA1/2* testing should be offered to all women younger than age 65 at the time of BC diagnosis, as well as for all female BC patients who are eligible for PARP inhibitor therapy, have TNBC, have a second contralateral or ipsilateral primary BC, or have a personal or family history suggestive of hereditary cancer. *BRCA1/2* testing is also recommended for all male BC patients. Finally, the guideline suggests that testing for HP genes beyond *BRCA1/2* should be offered to selected patients (30). However, the most recent ASCO guideline on GGT panels in patients with cancer proposes that when GGT is indicated for a patient with cancer, multigene panel testing should be offered if more than one gene is relevant, including *BRCA1*, *BRCA2*, *PALB2*, *CDH1*, *PTEN*, *STK11*, and *TP53* genes (31). In Brazil, we are far from implementing these guidelines, since GGT is not available in the public healthcare system, and in the private system, its coverage is only available for patients who fulfill restrictive criteria established by the ANS. Access to genetic testing is a challenge. Regulatory and policy actions, together with physician and patient education, are urgently needed to address these issues (32). This is a field of cancer care that should be substantially improved, since recent data revealed that only 26% of female patients with BC undergo GGT within the first 2 years after their diagnosis (33).

Our study demonstrated that the deleterious variants in HPM genes were more common in younger patients and in those with TNBC (14%) followed by HER2+ (9%) and HR+/HER2− (7%). Paixão et al. showed that the prevalence of P/LP variants in HMP was 22.8% in the TNBC subtype, 15.4% in HER2+, and 9.8% in HR+/HER2−. This study included high-risk patients with younger median age at diagnosis (44 years) and who met the NCCN criteria for GGT (18). The association between BC molecular subtype and germline findings is important not only from a testing perspective but also for interpretation of risk-reducing approaches. A clinical report suggests in some cases that germline pathogenic variants do not appear to play a major role in the tumorigenesis of BC (34). The CARRIERS study showed that the contralateral BC risk in *PALB2* carriers was only statistically higher in HR− patients, highlighting the importance of tumor phenotype in genetic counseling (35).

The adequate evaluation of germline status is critical for BC patients as patients who test positive for HP genes may be considered for risk-reducing strategies including increased surveillance, chemoprevention, and surgical interventions, alongside preventive measures in family members (36). Therefore, adequate evaluation of germline status should be incorporated into clinical practice as a predictive biomarker with important implications for optimal treatment of BC in the early (37) and advanced (38, 39) settings. Of note, the GGT results may help personalize risk-reducing strategies such as bilateral mastectomy in young patients with BC who test negative, since a recent study suggests a low 10-year cumulative incidence (2.2%) of second primary BC in this population (40).

The present analysis has some limitations. First, this study involved a laboratory cohort with paucity of clinical data including high-risk features such as family history of cancer, reproductive and gynecological history, and modified risk factors. Second, we had complete information in BC molecular subtypes only for ~30% of the cohort. Third, there was underrepresentation of some Brazilian regions in the scenario of genetic diversity in miscegenated populations. Finally, our cohort may not have been entirely unselected on the basis of guidelines’ criteria given that physicians may have ordered the testing based on common clinical criteria, such as age at diagnosis, BC subtype, and family history.

In conclusion, this study is the largest cohort from the perspective of the Brazilian private health system involving the germline profile of P/LP variants in HMP BC genes in a population who might not have been selected based on high-risk criteria. This study provided a broader understanding of germline BC genes and has the potential to open support regulatory actions, healthcare provider and patient education, and policy recommendations toward broader testing access. Therefore, GGT incorporation into routine practice should be strongly considered by healthcare providers in BC.

## Data Availability

The dataset and materials used to conduct this research is available from the authors upon request to interested researchers.

## In memoriam

All authors would like to dedicate this publication in memoriam of Bernardo Garicochea who passed away during this article submission, a remarkable mentor and leader of the Oncoclinicas cancer genetics team and a true visionary in genetics and genomics. His kindness, leadership, and support have left an indelible mark on our group, inspiring countless individuals to pursue new ideas and insights.
